# Usefulness of Xpert MTB/RIF Ultra to Rapidly Diagnose Sputum Smear-Negative Pulmonary Tuberculosis Using Bronchial Washing Fluid

**DOI:** 10.3389/fmicb.2020.588963

**Published:** 2020-09-22

**Authors:** Jung-Yien Chien, Ching-Kai Lin, Chong-Jen Yu, Po-Ren Hsueh

**Affiliations:** ^1^Department of Internal Medicine, National Taiwan University Hospital, National Taiwan University College of Medicine, Taipei, Taiwan; ^2^Department of Laboratory Medicine, National Taiwan University Hospital, National Taiwan University College of Medicine, Taipei, Taiwan

**Keywords:** pulmonary tuberculosis, Xpert MTB/RIF Ultra, COBAS TaqMan, MTB, bronchoscopy

## Abstract

This study evaluated the performance of the Xpert MTB/RIF Ultra assay (Xpert Ultra) to detect smear-negative pulmonary tuberculosis (PTB). Xpert Ultra assay was prospectively performed using bronchial washing fluid (BWF) in comparison to COBAS TaqMan MTB (COBAS) assay and mycobacterial culture. Of the 165 enrolled participants, 27 (16.4%) had PTB based on composite reference standard and 16 (9.7%) had culture-confirmed PTB. By the composite reference standard of PTB, the sensitivity of Xpert Ultra (63.0, 95% confidence interval, CI, 42.4–80.6%) was higher than the COBAS assay (25.9%, *P* = 0.006), BWF-culture (33.3%, *P* = 0.029) and sputum-culture (37.0%, *P* = 0.057). Meanwhile, the specificity of Xpert Ultra was 99.3% which was slightly lower than the 100.0% specificity of the COBAS assay (*P* = 1.000) and cultures (*P* = 1.000). Against the reference standard of culture-confirmed PTB, Xpert Ultra also had a higher sensitivity (62.5, 95% CI, 35.4–84.8%) than the COBAS assay (31.3%, *P* = 0.077) and was similar to BWF-culture (56.3%, *P* = 0.719) and sputum-culture (62.5%, *P* = 1.000). However, one subject with previously treated old PTB had a false-positive result on the Xpert Ultra assay. This prospective study showed Xpert Ultra assay using BWF had better sensitivity than COBAS assay and mycobacterial cultures but could represent a false positive in patients with inactive old PTB.

## Introduction

Tuberculosis (TB) caused by *Mycobacterium tuberculosis* (MTB) is the leading cause of deaths due to infectious disease worldwide (Chao et al., 2019; Huang et al., 2019). Early detection is essential for improving health outcomes of TB and reducing TB transmission. However, the delay in diagnosing TB and initiating treatment is often due to the lack of highly sensitive and rapid diagnostics (Uplekar et al., 2015; Liu et al., 2018; Leong et al., 2019; Lin et al., 2019). Acid-fast bacilli (AFB) smear microscopy is widely used to diagnose TB, yet its sensitivity is often low. Furthermore, MTB culture takes several weeks and thus cannot guide initial treatment decisions (Heemskerk et al., 2018). Therefore, patients who are smear-negative or sputum-scarce are often at an increased risk of a delay in their diagnosis, which is associated with poor outcomes. Several diagnostic nucleic acid amplification tests (NAATs) have been developed to reduce diagnostic gaps and delays in treatment.

The COBAS TaqMan MTB test (COBAS) (Roche Diagnostics, Basel, Switzerland) includes a dual-target approach for detection of MTB and is one of the most widely utilized NAATs for MTB detection (Park et al., 2013). The Xpert MTB/RIF assay (Xpert) (Cepheid, Sunnyvale, CA, United States), based on a nested real-time PCR, is a rapid, highly sensitive NAAT with the ability to perform additional rifampicin susceptibility testing (Boehme et al., 2010; Kuo et al., 2018). Meta-analyses of the diagnostic performance of Xpert for pulmonary TB (PTB) showed excellent sensitivity of 98% with smear-positive sputum, but the sensitivity of Xpert for PTB detection is suboptimal in patients with smear-negative sputum (Horne et al., 2019). Recently, the Xpert MTB/RIF Ultra assay (Xpert Ultra) (Cepheid, Sunnyvale, CA, United States) has been developed using two different multicopy amplification targets (IS6110 and IS1081) and a larger reaction chamber. This assay aims to improve the sensitivity for MTB detection and avoid false-positive rifampicin resistance results in patients with paucibacillary disease and has resulted in a 10-fold improvement in the lower limit of TB detection (Chakravorty et al., 2017).

Obtaining a high-quality specimen for testing is key to improve the accuracy of the diagnostic assay. Bronchoscopy is a useful tool for the diagnosis of PTB in patients who are smear-negative or those cannot produce sputum (Shin et al., 2012; Lee et al., 2013). Bronchoscopy specimens have been reported to be superior to induced sputum for the diagnosis of PTB (Charoenratanakul et al., 1995). However, the application of the Xpert Ultra assay in bronchial washing fluid (BWF) has not yet been evaluated. To understand these issues better, we prospectively examined the accuracy of the Xpert Ultra and COBAS assay performed with BWF obtained from smear-negative or sputum-scarce patients with suspected PTB recruited from a hospital in Taiwan compared to composite and reference standards for mycobacterial culture.

## Materials and Methods

### Study Participants and Inclusion Criteria

Altogether 165 adult subjects with suspected smear-negative PTB were prospectively enrolled between August 2018 and June 2019 at National Taiwan University Hospital (NTUH), a tertiary referral hospital in Taipei, Taiwan. All subjects underwent bronchoscopy and BWF were collected. Patient’s clinical data, including the medical history, radiologic and laboratory findings were prospectively collected. PTB was defined by a composite reference standard as either bacteriologically confirmed TB (culture-confirmed TB) or diagnosis with active TB by a clinician who has decided to give the patient a full course of TB treatment (clinically diagnosed TB) according to the World Health Organization’s definition (Eurosurveillance editorial Team, 2013). The study was approved by the institutional review board of National Taiwan University Hospital (201803019RIND).

### Laboratory Study

All BWFs were examined blindly by smear microscopy for AFB, by the COBAS and Xpert Ultra assays, and by cultures with both solid and liquid media. For the Xpert Ultra and COBAS assay, 0.5 ml BWF without decontamination or concentration was used and performed according to the manufacturer’s instructions. Briefly, samples were mixed with sample reagent of the Xpert Ultra kit for 15 min at room temperature and transferred into cartridge. The Xpert Ultra assay provides semi-quantitative results, “high,” “medium,” “low,” and “very low,” based on the cycle threshold of the *rpoB* probe and a “trace” category for samples tested positive for IS6110 and/or IS1081 detection in the absence of a signal of *rpoB* probes. The sample with invalid result was tested again and excluded from analysis if persistent invalid result. The remaining specimens were decontaminated and liquefied by NaOH–citrate–N-acetyl-L-cysteine at room temperature and concentrated. Acid-fast staining was performed with an auramine-rhodamine fluorescent stain, followed by confirmation with Ziehl-Neelsen staining and graded according to the American Thoracic Society guidelines. Processed samples were inoculated into two types of media: the BACTEC MGIT tube (Becton-Dickinson, Sparks, United States) and the Lowenstein-Jensen Medium slant (Becton-Dickinson, Sparks, United States). An immuno-chromatographic assay using mouse monoclonal antibodies to detect MPT64 protein, which is specific for MTB complex, was used in positive MGIT 960 cultures to distinguish MTB and Non-tuberculous Mycobacteria (SD TB Ag MPT 64 Rapid, Standard Diagnostics, Inc., South Korea). Positive cultures by MGIT tube were subcultured on 7H11 plates (Becton-Dickinson, Sparks, United States) and identified to the species level by a combination of morphology, growth rate of the colonies and biochemical tests. Susceptibility testing was performed using the indirect agar proportion method with a critical concentration of 1 μg/ml for rifampin (Kim, 2005).

### Statistical Analysis

Categorical variables were compared using a chi-square test or Fisher’s exact test, where appropriate, and differences in continuous variables were analyzed using a Student’s *t*-test. The data are presented as numbers (percentages), means ± standard deviations, or percentages (95% confidence intervals) unless otherwise noted. The statistical analyses were performed using STATA version 12 software (StataCorp LLC, TX, United States). Two-sided *P*-values of < 0.05 were considered statistically significant.

## Results

**FIGURE 1:**
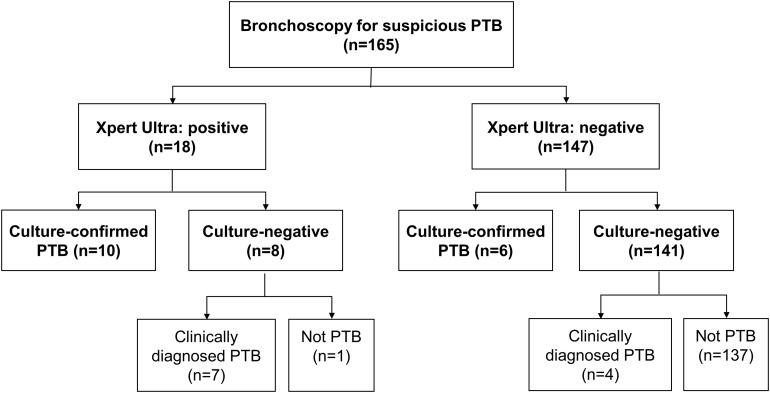
Flow diagram of the study participants. PTB, pulmonary tuberculosis.

**FIGURE 2:**
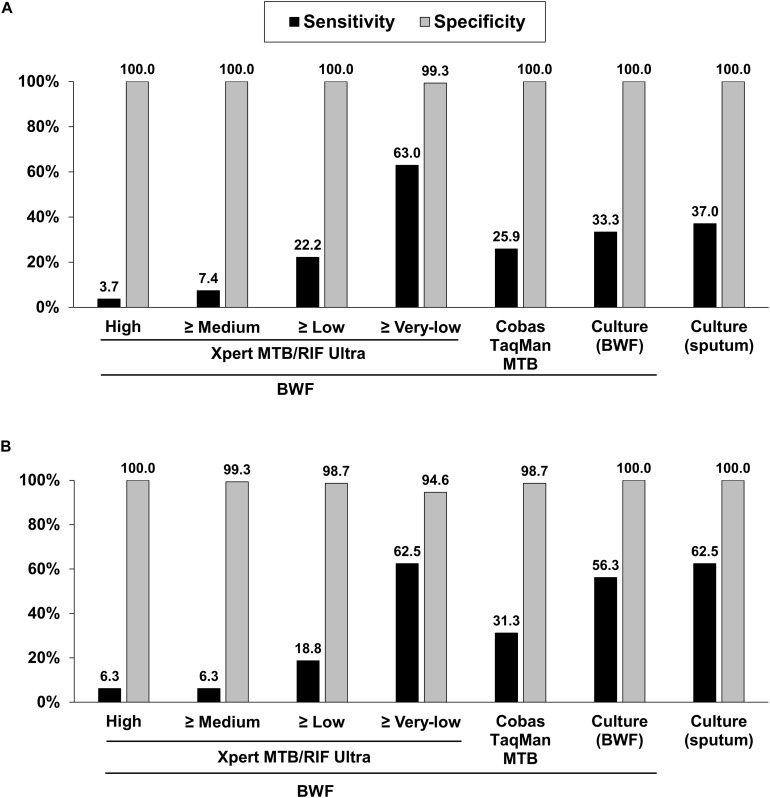
Sensitivities and specificities of diagnostic methods for pulmonary tuberculosis based on composite reference standards **(A)** and culture-confirmed pulmonary tuberculosis **(B)**. BWF, bronchial washing fluid.

Altogether 165 subjects undergoing bronchoscopy examination due to suspicion for PTB were enrolled. Their median age was 64 (24–95) years and 79 (47.9%) were male (Table 1). Of the 165 enrolled participants, 27 (16.4%) had PTB based on composite reference standard, 16 (9.7%) were culture-confirmed as PTB (10 subjects were sputum-culture positive and 9 were BWF-culture positive), 18 (10.9%) and 7 (4.2%) were positive on Xpert Ultra and COBAS assay, respectively, (Figures 1, 2). Table 1 shows the demographic data, comorbidity, and laboratory results, which were similar amongst the entire cohort.

**TABLE 1:** Characteristics of enrolled patients.

	Pulmonary tuberculosis*		Not pulmonary tuberculosis*	
**Characteristics**	**Culture-confirmed**	**Clinically diagnosed**		***P*-Value**
No. of patients	16	11	138	
Age (year)				0.083
<35	3 (18.8)	1 (9.1)	3 (2.2)	
35–49	3 (18.8)	2 (18.2)	19 (13.8)	
50–64	4 (25.0)	5 (45.5)	43 (31.2)	
65–79	4 (25.0)	2 (18.2)	60 (43.5)	
≧80	2 (12.5)	1 (9.1)	13 (9.4)	
Sex				0.467
Female	6 (37.5)	6 (54.5)	74 (53.6)	
Male	10 (62.5)	5 (45.5)	64 (46.4)	
Comorbidities				
Hypertension	1 (6.3)	1 (9.1)	30 (21.7)	0.223
Diabetes	3 (18.8)	1 (9.1)	17 (12.3)	0.714
Chronic obstructive pulmonary disease	1 (6.3)	1 (9.1)	8 (5.8)	0.907
Bronchiectasis	1 (6.3)	0 (0.0)	23 (16.7)	0.196
Chronic kidney disease	2 (12.5)	0 (0.0)	15 (10.9)	0.498
Location				0.669
Upper lobes	8 (50.0)	7 (63.6)	63 (45.7)	
Right middle lobe/Left lingula lobe	1 (6.3)	1 (9.1)	22 (15.9)	
Lower lobes	7 (43.8)	3 (27.3)	53 (38.4)	
Sputum				
Acid-fast bacilli smear (+)	0 (0.0)	0 (0.0)	0 (0.0)	–
Mycobacterial culture (+)	10 (62.5)	0 (0.0)	0 (0.0)	<0.001
Bronchial washing fluid				
Acid-fast bacilli smear (+)	1 (6.3)	1 (9.1)	0 (0.0)	0.005
Mycobacterial culture (+)	9 (56.3)	0 (0.0)	0 (0.0)	<0.001
Xpert MTB/RIF Ultra (+)	10 (62.5)	7 (63.6)	1 (0.7)	<0.001
High	1 (6.3)	0 (0.0)	0 (0.0)	
Medium	0 (0.0)	1 (9.1)	0 (0.0)	
Low	2 (12.5)	2 (18.2)	0 (0.0)	
Very low	7 (43.8)	4 (36.4)	1 (0.7)	
COBAS TaqMan MTB (+)	5 (31.3)	2 (18.2)	0 (0.0)	<0.001

*Pulmonary tuberculosis was diagnosed based on composite reference standard.

Table 2 show the correlation between different diagnostic methods. Among the 27 subjects with PTB, 10 (37.0%) and 9 (33.3%) were sputum-culture and BWF-culture positive, respectively, while 17 (63.0%) were positive on the Xpert Ultra assay. Among 18 subjects with Xpert Ultra positivity, one (5.6%), one (5.6%), four (22.2%), and twelve (66.7%) subjects had high, medium, low and very low positive results, respectively. Among those, 10 (55.6%) subjects had culture-confirmed PTB, 7 (38.9%) subjects had clinically diagnosed tuberculosis, and 1 (5.6%) with old PTB history had false-positive result in the Xpert Ultra test. Drug susceptibility tests and Xpert Ultra assay showed all MTB isolates were rifampicin susceptible. The turn-around time of the Xpert Ultra was 2 h, much shorter than COBAS assay (6 h) and the median time to positive of BWF-culture (42, 17–44 days).

**TABLE 2:** Correlation between diagnostic methods.

	Pulmonary Tuberculosis*					Not Pulmonary Tuberculosis*	
	**Results of Xpert MTB/RIF Ultra**					**Results of Xpert MTB/RIF Ultra**	
**Assay**	**High (*n* = 1)**	**Medium (*n* = 1)**	**Low (*n* = 4)**	**Very low (*n* = 11)**	**Negative (*n* = 10)**	**Very low (*n* = 1)**	**Negative (*n* = 137)**
**Culture**							
Positive, *n* (%)	1 (100.0)	0 (0.0)	1 (25.0)	6 (54.5)	1 (10.0)	0 (0.0)	0 (0.0)
Negative, *n* (%)	0 (0.0)	1 (100.0)	3 (75.0)	5 (45.5)	9 (90.0)	1 (100.0)	137 (100.0)
**COBAS TaqMan MTB**							
Positive, *n* (%)	1 (100.0)	1 (100.0)	1 (25.0)	4 (36.4)	0 (0.0)	0 (0.0)	0 (0.0)
Negative, *n* (%)	0 (0.0)	0 (0.0)	3 (75.0)	7 (63.6)	10 (100.0)	1 (100.0)	137 (100.0)

*Pulmonary tuberculosis was diagnosed based on composite reference standard.

Compared to PTB defined by the composite reference standard, the sensitivity, specificity, positive predictive value (PPV), and negative predictive value (NPV) of the Xpert Ultra test were 63.0, 99.3, 94.4, and 93.2%, respectively; whereas the corresponding values for COBAS were 25.9, 100.0, 100.0, and 87.3%, respectively; for BWF-culture were 33.3, 100.0, 100.0, and 88.5%, respectively; and for sputum-culture were 37.0, 100.0, 100.0, and 89.0%, respectively (Table 3 and Figure 2A). In comparison to the reference standard of culture-confirmed PTB, the sensitivity, specificity, PPV and NPV of the Xpert Ultra assay were 62.5, 94.6, 55.6, and 95.9%, respectively; whereas the corresponding values for COBAS were 31.3, 98.7, 71.4, and 93.0%, respectively; for BWF-culture were 56.3, 100.0, 100.0, and 95.5%, respectively, and for sputum-culture were 62.5, 100.0, 100.0, and 96.1%, respectively (Table 3 and Figure 2B).

**TABLE 3:** Performance of diagnostic methods.

**Definition of pulmonary tuberculosis**	**Assay**	**Performance% (95% confidence interval)**				
		**Sensitivity**	**Specificity**	**PPV**	**NPV**	**Accuracy**
Composite reference standard	Culture (sputum)	37.0 (19.4-57.6)	100.0 (97.4-100.0)	100.0 (69.2-100.0)	89.0 (83.0-93.5)	89.7 (84.0-93.9)
	Culture (BWF)	33.3 (16.5-54.0)	100.0 (97.4-100.0)	100.0 (66.4-100.0)	88.5 (82.4-93.0)	89.1 (83.3-93.4)
	COBAS (BWF)	25.9 (11.1-46.3)	100.0 (97.4-100.0)	100.0 (59.0-100.0)	87.3 (81.1-92.1)	87.9 (81.9-92.4)
	Xpert Ultra (BWF)	63.0 (42.4-80.6)	99.3 (96.0-100.0)	94.4 (72.7-99.9)	93.2 (87.8-96.7)	93.3 (88.4-96.6)
Culture-confirmed	Culture (sputum)	62.5 (35.4-84.8)	100.0 (97.6-100.0)	100.0 (69.2-100.0)	96.1 (91.8-98.6)	96.4 (92.3-98.7)
	Culture (BWF)	56.3 (29.9-80.2)	100.0 (97.6-100.0)	100.0 (66.4-100.0)	95.5 (91.0-98.2)	95.8 (91.5-98.3)
	COBAS (BWF)	31.3 (11.0-58.7)	98.7 (95.2-99.8)	71.4 (29.0-96.3)	93.0 (87.9-96.5)	92.1 (86.9-95.7)
	Xpert Ultra (BWF)	62.5 (35.4-84.8)	94.6 (89.7-97.7)	55.6 (30.8-78.5)	95.9 (91.3-98.5)	91.5 (86.2-95.3)

*BWF, bronchial washing fluid; CI, confidence interval; COBAS, COBAS TaqMan MTB; PPV, positive predictive value; NPV, negative predictive value; Xpert Ultra, Xpert MTB/RIF Ultra.*

Furthermore, when analyzing the sensitivity of the assays, compared to the composite reference standard for PTB, the sensitivity of the Xpert Ultra (63.0%) was higher than the COBAS assay (25.9%, *P* = 0.006), BWF-culture (33.3%, *P* = 0.029), and sputum-culture (37.0%, *P* = 0.057). Compared to the reference standard of culture-confirmed PTB, Xpert Ultra also had a higher sensitivity (62.5%) than the COBAS assay (31.3%, *P* = 0.077) and a similar sensitivity compared to BWF-culture (56.3%, *P* = 0.719) and sputum-culture (62.5%, *P* = 1.000). Meanwhile, compared to the composite reference standard, the specificity of Xpert Ultra was 99.3% which was only slightly lower than the 100.0% specificity of the COBAS assay (*P* = 1.000) and cultures (*P* = 1.000). However, when compared to culture-confirmed PTB, Xpert Ultra had lower specificity (94.6%) than the COBAS assay (98.7%, *P* = 0.103) and cultures (100.0%, *P* = 0.007). These findings resulted in a lower false-negative rate in the Xpert Ultra assay (*n* = 10, 37.0%) than sputum-culture (*n* = 17, 63.0%), BWF-culture (*n* = 18, 66.7%) and COBAS assay (*n* = 20, 74.1%; *P* = 0.032) and a slightly higher false-positive rate in the Xpert Ultra assay.

## Discussion

Challenges to the rapid diagnosis of paucibacillary PTB include negative smear microscopy results and the long incubation time for MTB culture. In this pilot study, 165 consecutive BWFs were processed simultaneously using fluorescent smear microscopy, mycobacterial cultures with both solid and liquid media, and the COBAS and Xpert Ultra assays and the performances were evaluated against composite reference standards and culture-confirmed reference standards. Against the composite reference standard of PTB, the sensitivity of Xpert Ultra (63.0%) was much higher than the COBAS assay (25.9%), BWF-culture (33.3%), and sputum-culture (37.0%). Meanwhile, the specificity of Xpert Ultra was 99.3%, which was only slightly lower than the 100.0% specificity of COBAS assay and cultures. For diagnosis of culture-confirmed PTB, Xpert Ultra assay also had a higher sensitivity (62.5%) than the COBAS assay and was comparable to BWF-culture (56.3%) and sputum-culture (62.5%). However, Xpert Ultra had a lower specificity (94.6%) than the COBAS assay (98.7%) and cultures (100.0%). To our knowledge, no study has been undertaken to compare the Xpert Ultra, the COBAS assay and cultures against composite and culture-confirmed reference standard, making this the first.

In our study, the sensitivity of Xpert Ultra among BWF was 63.0%, which was similar to the results in a multicenter study in patients with paucibacillary disease (smear-negative, culture-positive) and in patients with HIV (Dorman et al., 2018). Among 27 PTB patients diagnosed by composite reference standards, we found 18 (66.7%) patients were BWF-culture negative (11 had clinically diagnosed PTB and 7 were only sputum-culture positive, Figure 1). Among those 18 subjects, the Xpert Ultra assay yielded positive results in 10 (55.6%) patients. In addition, among 16 of the 165 patients with culture-confirmed PTB, we found 2 (12.5%) patients had positive Xpert Ultra and negative BWF-culture results. This suggests that the Xpert Ultra assay might be more sensitive than mycobacterial culture in some situations. The high sensitivity of the Xpert MTB/RIF assay could be attributed to the additional MTB-specific hybridization targets (insertion sequences IS1081 and IS6110) and a larger reaction chamber to improve the sensitivity of Xpert Ultra with a lower limit of MTB detection of about 16 CFU/ml (Chakravorty et al., 2017). In addition, the Xpert Ultra and the COBAS assay were performed with direct specimens, while the culture used decontaminated sediments, which may result in a loss of MTB during the process.

Our study also highlights the importance of bronchoscopy in the diagnosis of paucibacillary PTB. Only 10 of 27 PTB patients were confirmed with sputum-culture, but BWF from bronchoscopy yielded positive results in 23 of the 27 PTB patients. Of the 13 patients, 1 was positive only on BWF-culture, 7 were positive on Xpert Ultra assay, and 5 were positive on both BWF-culture and Xpert Ultra assay, with increasing sensitivity from 37.0 to 85.2%. Meanwhile, only 10 of 16 culture-confirmed PTB patients were confirmed by sputum-culture, with the Xpert Ultra assay of BWF identifying 5 more patients with PTB (sensitivity increased from 62.5 to 93.8%). However, in our study, the sensitivity of Xpert Ultra with BWF (63.0%) was lower than that previously reported with Xpert Ultra on bronchial alveolar lavage fluid (BALF, 93%) (Theron et al., 2013). Among the 16 culture-confirmed PTB patients, 6 (37.5%) had false-negative results on Xpert Ultra with BWF (one was BWF-culture positive and 5 were sputum-culture positive only) and this might imply lower sensitivity of the Xpert Ultra with BWF compared to BALF. However, transient hypoxia might occur more frequently during BAL procedures (Cole et al., 1980) and could lead to higher rate of respiratory failure in patients with chronic pulmonary disease. It should be taken into consideration that BWF from bronchoscopy could be safer in clinical practice.

Recently, a meta-analysis reported that the higher sensitivity of Xpert Ultra comes at the expense of a loss of specificity (Zhang et al., 2020). Although highly sensitive, the lower specificity of the Xpert Ultra was a cause for concern as it can lead to false positives and might put such patients on toxic anti-TB regimens (Osei Sekyere et al., 2019). The specificity of Xpert Ultra in our study was 99.3% when the composite reference standards were used, but the specificity decreased slightly to 94.6% when culture-confirmed PTB was used as a reference standard. Compared to the composite reference standard, when mycobacterial culture was used as the reference standard, a previous study also showed reduced specificity in Xpert Ultra (Dorman et al., 2018). While the culture has an analytical sensitivity threshold of 10 colony forming units per ml, the decontamination step could reduce the number of bacilli in the specimen and make culture an imperfect gold standard. Therefore, final diagnosis may be more accurately performed with a composite evaluation consisting of radiological findings, microbiological results, and treatment response. When culture is used as a gold standard, this limitation makes it difficult to provide a true specificity of the NAATs. However, it is worthwhile to note that the culture assay cannot identify dead and non-viable MTB, a situation that can lead to false-positive results since the Xpert Ultra can detect DNA in samples (Osei Sekyere et al., 2019). In our study, among 138 patients without PTB, one (0.7%) patient with old PTB history had a false-positive result in the Xpert Ultra assay.

This study has several limitations. First, although its use is increasing, bronchoscopy is not yet widely available in resource-poor settings. Second, the number of patients with PTB in this study was relatively small. Third, we did not include samples from healthy patients to serve as negative controls. Finally, the performance of Xpert Ultra assay on detection of rifampin resistance could not be evaluated because there were no case with rifampin resistance on any of the assays.

The Xpert Ultra assay using BWF showed better sensitivity than the COBAS assay and mycobacterial cultures and performed within a faster timeframe. In addition, the specificity was high with only one false-positive case by the Xpert Ultra assay from a patient with a history of PTB that was negative by both cultures and the COBAS assay. Because Xpert Ultra can provide semi-quantitative information on the bacterial load of specimens (Theron et al., 2011, 2012) the results of Xpert Ultra could be also helpful to assess disease severity (Perrin et al., 2010), stratify patients likely to be highly infectious and identify those at risk of treatment failure (Hesseling et al., 2010; Bark et al., 2012; Visser et al., 2012). However, Xpert Ultra still did not have a sufficiently high negative predictive value to rule out PTB.

## Data Availability Statement

The raw data supporting the conclusions of this article will be made available by the authors, without undue reservation, to any qualified researcher.

## Ethics Statement

The studies involving human participants were reviewed and approved by the institutional review board of National Taiwan University Hospital (201803019RIND). The ethics committee waived the requirement of written informed consent for participation.

## Author Contributions

J-YC and P-RH assumed responsibility for the content of the manuscript, including the data and analysis. C-KL and C-JY had full access to all study data and assumed responsibility for the integrity of the data and the accuracy of the data analysis. J-YC, C-KL, and P-RH contributed substantially to the study design, data analysis and interpretation, and the writing of the manuscript. All authors contributed to the article and approved the submitted version.

## Conflict of Interest

The authors declare that the research was conducted in the absence of any commercial or financial relationships that could be construed as a potential conflict of interest.
